# Drug Use and Social Connectivity Related to Hepatitis C Infection Among Rural People Who Use Drugs

**DOI:** 10.1111/jvh.70200

**Published:** 2026-06-11

**Authors:** Alex Rains, Ellen Almirol, William Nicholson, Scott Fletcher, Wiley D. Jenkins, John A. Schneider, Mai T. Pho

**Affiliations:** ^1^ Department of Psychiatry Brigham and Women's Hospital Boston Massachusetts USA; ^2^ Chicago Center for HIV Elimination University of Chicago Chicago Illinois USA; ^3^ The Southern Illinois Resource and Advocacy Center Shawneetown Illinois USA; ^4^ Department of Public Health Sciences Clemson University Clemson South Carolina USA; ^5^ Department of Medicine University of Chicago Chicago Illinois USA; ^6^ Department of Public Health Sciences University of Chicago Chicago Illinois USA

**Keywords:** hepatitis, rural environment, social networks

## Abstract

People who use drugs (PWUD) have a disproportionately elevated risk of hepatitis C virus (HCV). Appropriate screening and treatment may be limited, especially in rural environments experiencing healthcare shortages. In order to develop targeted intervention strategies for rural PWUD, we investigated individual and network‐level correlates of HCV positivity. Survey data were collected from PWUD from the Illinois region of the Federal Delta Regional Authority (DRA). The primary outcome of interest was HCV positivity by self‐report. We explored associations between HCV positivity and individual demographic, drug use, and service utilisation characteristics as well as degree of connectedness within social networks. Three hundred and two participants were included in analyses. At the individual level, HCV positivity was associated with injection drug use, overdose history, and prior receipt of substance use treatment. Two hundred and one participants had at least one connection to a peer via referral chains, most of which contained a mix of HCV‐negative and positive individuals. Of note, isolated individuals had comparable rates of HCV positivity compared to their socially connected peers. Degree of connectedness was not associated with HCV positivity. This study highlights the heterogeneity, both with respect to HCV positivity and other characteristics, among networks of rural PWUD. Given that most participants were connected to others, HCV interventions led by peer champions may facilitate improved access to critical services for risk mitigation.

## 1 Introduction

Hepatitis C virus infection (HCV) poses a substantial public health burden, largely due to the morbidity and mortality effects of resultant liver disease. At especially high risk are people who use drugs (PWUD), who are estimated to have an HCV prevalence between 40% and 70% at the population level [1, 2, 3]. While HCV infection is curable, testing and treatment are frequently limited in some communities and among certain populations. Often, rural locales are most vulnerable to outbreaks of bloodborne illnesses including HCV [4]. With disproportionately rising rates of HCV in rural counties [5], and ongoing closures of numerous rural hospitals that may have previously provided care for patients with HCV [6], rural communities will need to prioritise HCV prevention, testing, and early treatment efforts in order to minimise impacts of HCV‐related morbidity and mortality on their communities.

Despite recognition of the disproportionately elevated risk of HCV among PWUD, testing and treatment for this community remain limited across settings. In particular, availability of HCV services is limited in rural settings, which are often unable to access healthcare due to transportation difficulties and provider shortages, especially those who offer care for substance use and related health issues [7, 8, 9, 10, 11]. In addition, the rapidly changing landscape of rural substance use, now characterised by a marked increase in the use of stimulants including methamphetamine, may further influence rural HCV transmission, for reasons such as engagement in higher‐risk sexual behaviours and injection equipment sharing among people who use methamphetamine [12, 13, 14, 15, section 1; 16]. Furthermore, the social environment in some rural locales may be characterised by more frequent equipment‐sharing (especially in areas where access to sterile syringes is limited), perpetuation of incorrect information about HCV within networks, and smaller, more intimately connected PWUD networks than in urban areas [11]. All of these factors contribute to the rural risk environment, that is, the intersection of economic, physical, and structural determinants of drug‐related harms specific to rural milieus [17, 18, 19].

HCV infection among PWUD may be common in certain social and drug use networks [17, 20, 21]. Investigations at the network level can provide insights into risk distribution and facilitate identification of candidate networks for tailored interventions. However, despite the unique structural vulnerabilities and high risk for HCV infection among rural PWUD [4, 5, 22], literature specific to rural network characteristics remains limited. The few existing studies of rural network‐level HCV correlates [23, 24] have identified associations between network characteristics such as centrality, density, and the presence of high‐risk ties and HCV positivity. While studies of infection transmission within urban and suburban drug use networks [25, 26, 27] may have findings applicable to rural PWUD, they may not effectively capture social dynamics specific to the rural risk environment.

Given the paucity of up‐to‐date social network information of PWUD in highly vulnerable rural areas, further inquiry is needed to identify network traits that may help inform evidence‐based, locally tailored interventions to mitigate HCV transmission. The aim of the present study was to characterise rural networks of PWUD through a field‐based evaluation of peer referral chains, assessing HCV prevalence and network characteristics in order to improve linkage to prevention, testing, and treatment interventions for this group.

## 2 Methods

### 2.1 Setting and Study Population

The Ending Transmission of HIV, Hepatitis C, Sexually Transmitted Diseases and Overdose in Rural Communities of People Who Inject Drugs (“ETHIC”) trial drew participants from the 16 southernmost counties in Illinois, a region comprising the Illinois component of the Federal Delta Regional Authority (DRA). The DRA comprises over 200 counties and parishes in states along the Mississippi River which are primarily rural and driven by agricultural industries [28]. ETHIC was a site of The Rural Opioid Initiative (ROI), a federally funded consortium of 8 studies spanning 10 states and 65 counties in the U.S. aimed at understanding the opioid overdose and infectious disease syndemic and developing tailored interventions for PWUD in rural areas. Eligibility requirements for ETHIC participation included age of 15 years or older, residence in the Illinois counties of the DRA, and English fluency. Initial participants (i.e., those recruited beginning August 2020) were also required to have used opioids non‐medically by any route and/or injected any illicit drugs in the past 30 days; subsequently (i.e., beginning January 2022), eligibility criteria were expanded to also include people who used stimulants by any route (not solely injection) in the past 30 days prior to their study visit [29]. This project received ethics review and approval from the Institutional Review Board at the University of Chicago (protocol number IRB 17‐1630).

### 2.2 Data Collection

Participants were recruited from August 2020 to June 2023. Recruitment was initiated through a convenience sample of participants recruited by local partners (including harm reduction organisations, health departments, and community health agencies) and through street outreach. Additionally, incentivised peer referral allowed study participants to refer individuals in their social network, with compensation ($20) for each referral who was eligible for the study regardless of whether they consented to participate [30].

Following completion of written, informed consent, participants were administered surveys that captured sociodemographic data, drug of choice, overdose experiences, injection drug use, engagement in harm reduction services, HCV testing and treatment, and other healthcare experiences.

### 2.3 Study Outcomes

Our primary outcome of interest was HCV positivity by self‐report. Participants also reported other steps in the HCV care cascade: if they had taken or completed a course of medicine to treat their HCV; and if they had ever been told that their HCV was cleared or cured. Since HCV positivity by self‐report necessitated individuals having previously received testing for HCV, it should be noted that this measure generated more nuanced data than other measures of HCV positivity may have. This is expanded upon further in the Results and Discussion sections.

We evaluated characteristics of referral networks within our sample. Specific attention was given to identifying characteristics of those participants with a peer enrollment degree of one or more (e.g., peer referrers) and those who were not part of referral chains. We compared “isolates” (defined as individuals who were neither referred into the study, nor had referred anyone into the study) of our sample with those who were “non‐isolates” (e.g., referred in by others or who made referrals). The choice to operationalise this into a binary variable instead of stratifying participants by degree centrality, as is often the case in network analysis, was made deliberately. The average degree connectedness of our sample was less than 1, therefore comparing individuals by degree centrality would yield very small subsamples for those with higher degree centrality. Thus, we chose to compare those with a degree centrality of 0 against those with a degree centrality of 1 or more.

We also compared those with an enrollment degree of 1 or more with those who had an out‐degree of 0. In other words, we examined characteristics of “referrers,” who were defined as having made a referral that led to a peer's enrollment in the study, compared to those who were “non‐referrers.” Finally, we explored whether the number of first‐degree connections to other PWUD was associated with HCV positivity.

### 2.4 Statistical Analysis

Participants who were enrolled in the ETHIC study and responded to “Have you ever been told you had hepatitis C?” either with a yes or no were included in the analysis. If the response was “I don't know” (*n* = 3) or missing (*n* = 1), these participants were excluded from this analysis. Bivariate analysis was conducted between participants who reported they were diagnosed with HCV and who did not report viral clearance (i.e., who self‐reported current HCV positivity) with those who did not have HCV or had previously cleared HCV. We also examined differences between social groups (e.g., “isolates” versus “non‐isolates” and “referrers” versus “non‐referrers”) were tested using *t*‐test for continuous variables and *χ* ^2^ test for categorical variables.

Statistical significance was indicated with a *p*‐value less than or equal to 0.05. Analyses were conducted using STATA (Software Version 16, [31]) and R Statistical Software (v4.1.2; [32]). Gephi [33] and UCINet [34] were used for visualisation of social network data.

## 3 Results

### 3.1 Sociodemographic Characteristics

A total of 302 participants were included in the analysis (Table 1). Participants' average age was 42 (SD 11.3) years old, 64.6% were male, and 75.5% identified as white. Among the total sample, 209 (69.2%) participants reported current methamphetamine use, while 100 (33.1%) reported current opioid use. A majority of participants (65.2%) reported injection drug use in the past 30 days. Nearly half (41%) had experienced an overdose in the past. Regarding service usage, most (87.4%) participants had medical insurance, while almost half (46.7%) were currently connected with a primary healthcare provider. Many participants (65.9%) had previously accessed treatment for substance use, but only about one‐third of the sample (30.5%) reported having accessed harm reduction services in the 6 months preceding the study visit.

**TABLE 1:** Sample characteristics, stratified by hepatitis C virus (HCV) status^a^ (*N* = 302).

	Total (*n* = 302)^a^	HCV positive (*n* = 76)	HCV negative (*n* = 226)	*p*
	*N* (%)	*N* (%)	*N* (%)	
*Sample characteristics*				
Age, mean (SD)	42.0 (11.3)	42.3 (10.8)	41.9 (11.5)	0.774
Gender, male	195 (64.6%)	51 (67.1%)	144 (63.7%)	0.080
Race^b^				
White	228 (75.5%)	70 (92.1%)	158 (69.9%)	**0.002**
Black	54 (17.9%)	5 (6.6%)	49 (21.7%)	
Mixed race	14 (46.4%)	1 (1.3%)	13 (5.8%)	
Other/Uncertain	6 (2.0%)	0 (0%)	6 (2.7%)	
Arrested, past 6 months	97 (32.1%)	26 (34.2%)	71 (31.4%)	0.652
Unhoused, past 6 months	180 (59.6%)	41 (53.9%)	139 (61.5%)	0.305
*Drug use characteristics*				
Current drug use				
Methamphetamine	209 (69.2%)	54 (71.1%)	155 (68.6%)	0.795
Opioids^c^	100 (33.1%)	34 (44.7%)	66 (29.2%)	**0.020**
Multiple drugs	100 (33.1%)	25 (32.9%)	75 (33.2%)	0.963
IDU, past 30 days	197 (65.2%)	67 (88.2%)	130 (57.5%)	**< 0.001**
Past overdose	124 (41.1%)	42 (55.3%)	82 (36.3%)	**0.004**
*Service use characteristics*				
Has medical insurance	264 (87.4%)	67 (88.2%)	197 (87.2%)	0.822
Currently seeing a PCP	141 (46.7%)	37 (48.7%)	104 (46.0%)	0.787
Past treatment for substance use	199 (65.9%)	62 (81.6%)	137 (60.6%)	**0.001**
Accessed HRS past 6 months	92 (30.5%)	35 (46.1%)	57 (25.2%)	**0.001**
Access to transportation for appointments	261 (86.4%)	67 (88.2%)	194 (85.8%)	0.610
*Social characteristics*				
First‐degree connections, mean (SD)	1 (1.2)	1.1 (1.4)	1 (1.1)	0.524
Isolates^d^	101 (33.4%)	24 (36.9%)	77 (31.6%)	0.800
Referrer^e^	67 (22.2%)	14 (18.4%)	53 (23.5%)	0.451

*Note:* Bold indicates a *p*‐value < 0.05.

Abbreviations: HRS, harm reduction services; IDU, injection drug use; PCP, primary care physician; SD, standard deviation.

^a^ Total sample excluded 4 participants who responded to HCV questions with “I don't know” or missing response.

^b^ Race analysed as white vs. non‐white given small counts for other groups.

^c^ Opioids = heroin, fentanyl, prescription opioids, methadone, buprenorphine.

^d^ Isolates = individuals who were neither referred into the study nor had referred anyone into the study.

^e^ Referrers = individuals who made a referral that led to a peer's enrollment in the study.

### 3.2 HCV Characteristics

A total of 78% (*n* = 235/302) had ever been tested for HCV before their survey, with a quarter of participants (25%, *n* = 76/302) ever being told that they had HCV. Of those who reported having had HCV, 25% (*n* = 19/76) reported having taken or completed medicine to treat their HCV. 40% (*n* = 29/73) had ever been told that their HCV was cleared or cured. While all study participants were offered HCV screening after study enrollment, only 263 of the 302 accepted testing, and among those who screened positive (*n* = 45), even fewer (*n* = 9) underwent confirmatory testing, limiting the ability to draw conclusions from this data.

We compared the individual‐level characteristics of those who were HCV‐positive and HCV‐negative by self‐report, as described in the Methods section (Table 1). On average, the HCV‐positive group had a greater proportion of non‐Hispanic white participants, as opposed to Black, mixed race, or Hispanic participants (92.1% vs. 69.9%, *p* < 0.01). HCV‐positive participants reported opioids as their primary drug of choice at a greater rate than those who were HCV‐negative (44.7% vs. 29.2%, *p* = 0.02). A greater proportion of the HCV‐positive group reported injection drug use in the last 30 days (88.2% vs. 57.5%, *p* < 0.01) and a history of drug overdose in the past (55.3% vs. 36.3%, *p* < 0.01). A greater proportion of the HCV‐positive group received past treatment for substance use than participants who were HCV‐negative (81.6% vs. 60.6%, *p* < 0.01). More HCV‐positive participants accessed harm reduction services in the 6 months prior to survey administration compared to HCV‐negative participants (46.1% vs. 25.2%, *p* < 0.01). There was no significant difference in access to insurance, primary care, or transportation for appointments between groups.

### 3.3 Social Connectivity Characteristics

We identified 101 “isolates” in our sample (Table 2). With respect to drug use characteristics, these participants were less likely to report current methamphetamine use compared to their non‐isolated peers (60.4% vs. 73.6%, *p* = 0.03). They were also less likely to report 30‐day injection drug use (53.5% vs. 71.1%, *p < 0*.01) and a history of overdose (32.7% vs. 45.3%, *p* = 0.04). Isolates were less likely to report arrest within the past 6 months (23.8% vs. 35.8%, *p* = 0.03). Isolates and non‐isolates did not differ by HCV positivity.

**TABLE 2:** Sample characteristics, stratified by isolate status (*N* = 302).

	Total (*n* = 302)	Isolates (*n* = 101)	Non‐isolates (*n* = 201)	*p*
	*N* (%)	*N* (%)	*N* (%)	
*Sample characteristics*				
Age, mean (SD)	42.0 (11.3)	43.8 (11.8)	41.1 (10.9)	**0.051**
Gender, male	195 (64.6%)	66 (65.3%)	129 (64.2%)	0.841
Race^a^				
White	228 (75.5%)	69 (68.3%)	159 (79.1%)	0.100
Black	54 (17.9%)	29 (28.7%)	25 (12.4%)	
Mixed race	14 (46.4%)	6 (5.9%)	8 (4.0%)	
Other/Uncertain	6 (2.0%)	1 (1%)	5 (2.5%)	
Arrested, past 6 months	97 (32.1%)	24 (23.8%)	72 (35.8%)	**0.034**
Unhoused, past 6 months	180 (59.6%)	54 (53.5%)	126 (62.7%)	0.157
*Drug use characteristics*				
Current drug use				
Methamphetamine	209 (69.2%)	61 (60.4%)	148 (73.6%)	**0.027**
Opioids^b^	100 (33.1%)	34 (33.7%)	66 (32.8%)	0.990
Multiple drugs	100 (33.1%)	36 (35.6%)	64 (31.8%)	0.594
IDU, past 30 days	197 (65.2%)	54 (53.5%)	143 (71.1%)	**0.002**
Past overdose	124 (41.1%)	33 (32.7%)	91 (45.3%)	**0.036**
*Service use characteristics*				
Has medical insurance	264 (87.4%)	89 (88.1%)	175 (87.1%)	0.850
Currently seeing a PCP	141 (46.7%)	46 (45.5%)	115 (57.2%)	0.073
Past treatment for substance use	199 (65.9%)	61 (60.4%)	138 (68.7%)	0.194
Accessed HRS past 6 months	92 (30.5%)	31 (30.7%)	61 (30.3%)	0.951
Access to transportation for appointments	261 (86.4%)	87 (86.1%)	174 (86.6%)	0.061

*Note:* Bold indicates a *p*‐value < 0.05.

Abbreviations: HRS, harm reduction services; IDU, injection drug use; PCP, primary care physician; SD, standard deviation.

^a^ Race analysed as white vs. non‐white given small counts for other groups.

^b^ Opioids = heroin, fentanyl, prescription opioids, methadone, buprenorphine.

We identified 67 individuals as “referrers” (Table 3). Compared to their non‐referrer peers, these participants were significantly less likely to report current use of multiple drugs of choice (20.9% vs. 36.6%, *p* = 0.02). They were more likely to report 30‐day injection drug use (76.1% vs. 62.1%, *p* = 0.03) and a history of accessing harm reduction services in the last 6 months (43.3% vs. 26.8%, *p* = 0.02). Referrers and non‐referrers did not have significantly different rates of HCV positivity by self‐report.

**TABLE 3:** Sample characteristics, stratified by referrer status (*N* = 302).

	Total (*n* = 302)	Referrers (*n* = 67)	Non‐referrers (*n* = 235)	*p*
	*N* (%)	*N* (%)	*N* (%)	
*Sample characteristics*				
Age, mean (SD)	42.0 (11.3)	40.1 (11.4)	42.5 (11.2)	0.133
Gender, male	195 (64.6%)	40 (59.7%)	155 (70%)	0.345
Race^a^				
White	228 (75.5%)	58 (86.6%)	170 (72.3%)	0.084
Black	54 (17.9%)	8 (11.9%)	46 (19.6%)	
Mixed race	14 (46.4%)	1 (1.5%)	13 (5.5%)	
Other/Uncertain	6 (2.0%)	0 (0%)	6 (2.6%)	
Arrested, past 6 months	97 (32.1%)	23 (34.3%)	74 (31.5%)	0.661
Unhoused, past 6 months	180 (59.6%)	41 (61.2%)	139 (59.1%)	0.873
*Drug use characteristics*				
Current drug use				
Methamphetamine	209 (69.2%)	50 (74.6%)	159 (67.7%)	0.347
Opioids^b^	100 (33.1%)	20 (29.9%)	80 (34%)	0.620
Multiple drugs	100 (33.1%)	14 (20.9%)	86 (36.6%)	**0.020**
IDU, past 30 days	197 (65.2%)	51 (76.1%)	146 (62.1%)	**0.034**
Past overdose	124 (41.1%)	31 (46.3%)	93 (39.6%)	0.234
*Service use characteristics*				
Has medical insurance	264 (87.4%)	62 (92.5%)	202 (86%)	0.279
Currently seeing a PCP	141 (46.7%)	32 (47.8%)	109 (46.4%)	0.952
Past treatment for substance use	199 (65.9%)	48 (71.6%)	151 (64.3%)	0.328
Accessed HRS past 6 months	92 (30.5%)	29 (43.3%)	63 (26.8%)	**0.015**
Access to transportation for appointments	261 (86.4%)	60 (89.6%)	201 (85.5%)	0.397

*Note:* Bold indicates a *p*‐value < 0.05.

Abbreviations: HRS, harm reduction services; IDU, injection drug use; PCP, primary care physician; SD, standard deviation.

^a^ Race analysed as white vs. non‐white given small counts for other groups.

^b^ Opioids = heroin, fentanyl, prescription opioids, methadone, buprenorphine.

Finally, we explored whether the number of first‐degree connections (e.g., PWUD who the individual either referred into the study, or by whom the individual was referred into the study) was associated with HCV positivity. The mean number of first‐degree connections was quite low in our sample, with an average of one connection per person. There were no differences in the number of connections between HCV positive and HCV negative groups.

**FIGURE 1:**
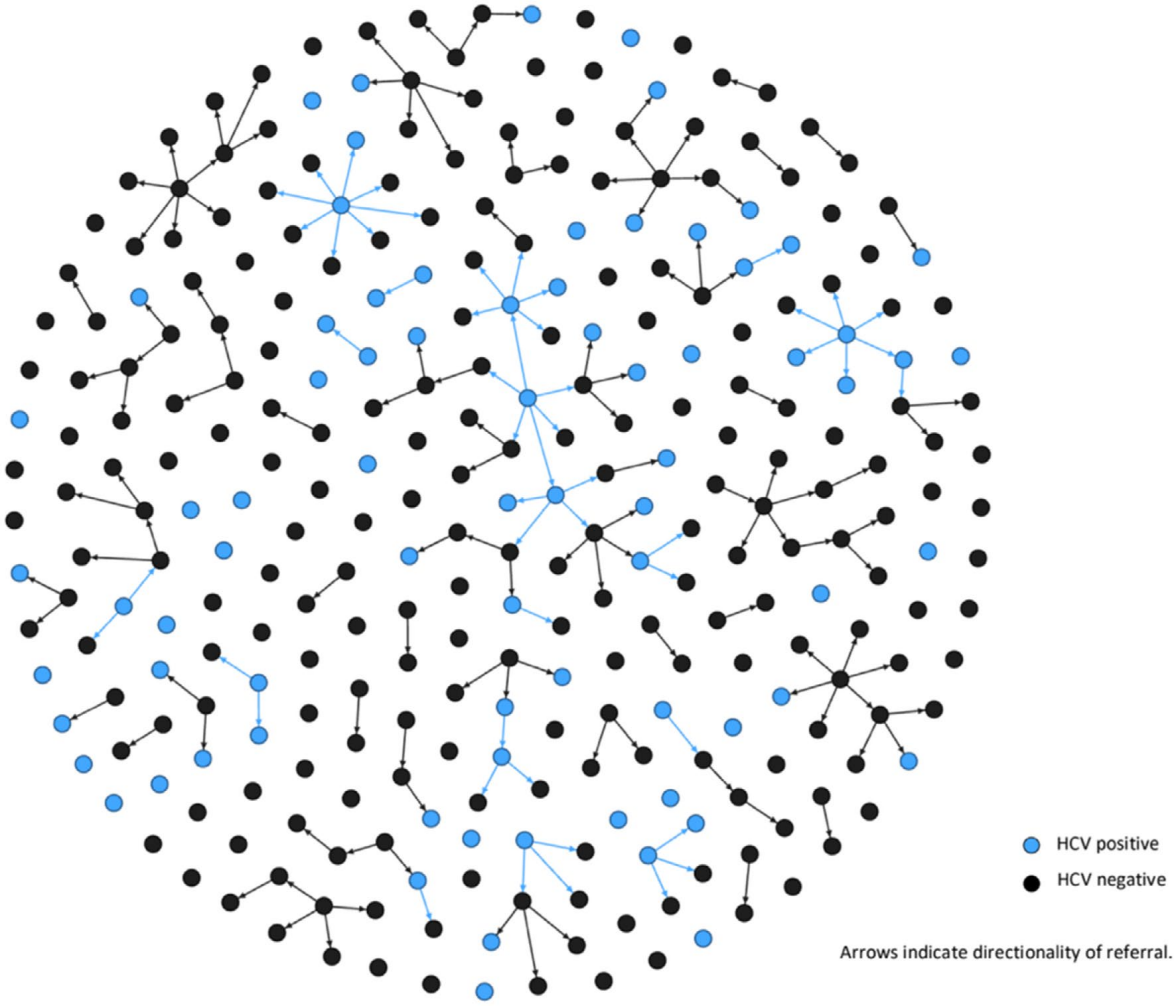
Referral chains by HCV status.

Referral chains and proportions of isolated versus non‐isolated participants are visually depicted in Figure 1. Blue and black nodes represent those individuals who self‐reported HCV positive and HCV negative, respectively. Arrows indicate the direction of a peer‐to‐peer referral. Of the 43 referral chains depicted, 21 contain a mix of HCV positive and HCV negative individuals.

## 4 Discussion

This study highlights network characteristics relevant to implementation of HCV testing and treatment among rural PWUD. We identified heterogeneity in HCV status within referral chains, with HCV‐positive and HCV‐negative participants interspersed throughout the personal networks, as visualised in Figure 1, and overall short referral chains. This study also highlights relationships between several individual‐level characteristics and HCV positivity, which both bolster the existing literature and serve as starting points for identifying high‐risk populations for tailored HCV prevention and treatment interventions.

Consistent with many prior studies of sociodemographic correlates of HCV, we identified an association of HCV positivity with white race [35, 36, 37]. Given that the large majority of participants were white, this may reflect a sampling issue and should be interpreted with caution. Notably, other studies have demonstrated greater HCV prevalence among Black populations [38, 39]. Whether our finding reflects a true difference in our rural setting, versus an underreporting among non‐white participants in our sample, versus a sampling issue, remains unclear. With respect to drug use behaviours, injection drug use was associated with HCV, as expected based on transmissibility of the virus through injection supplies [40, 41, 42, 43]. Also consistent with prior literature were our findings that HCV positivity was associated with a history of overdose [35, 44] and primary use of opioids as an individual's drug of choice [45]. These findings support the conceptualisation of opioid use, overdose, and HCV as a syndemic of diseases emerging under shared social contexts and concentrating among particular populations [37, 46, 47].

Finally, we identified a relationship between HCV positivity and prior treatment for substance use. A recent study examining data from the Rural Opioid Initiative as a whole noted a similar correlation, identifying a correlation between HCV positivity and utilisation of long‐acting medications for opioid use disorder [48]. There are multiple possible explanations for this finding. First, it may be that those with drug use characteristics that carry an elevated risk of HCV infection (e.g., injection drug use versus non‐injection drug use, duration of injection during the lifetime) access substance use treatment at greater rates. Second, treatment for substance use itself may facilitate increased uptake of HCV testing, whether through referrals or on‐site testing. Finally, people able to access healthcare, including substance use treatment, may have higher utilisation of HCV testing opportunities because they access healthcare services as a whole more frequently. We discuss opportunities for interventions based on this finding below.

Social connectivity data provided further insights into our study population. Given the role of recruiters/referrers and their network associates in prior studies of social network interventions [49], we chose to examine characteristics associated with each individual's level of connection to their peers. Most participants in our sample were connected to at least one other participant, either through referring them to the study or vice versa. Isolated individuals were less likely to report primary methamphetamine use, injection drug use, prior overdose, and arrest history. Of note, a number of isolates did report HCV positivity, suggesting that despite this categorisation, they were likely to have been connected to others at some point in time, though perhaps in ways that were more difficult to identify for the sake of study. Referrers reported less polysubstance use and higher rates of injection drug use and receipt of harm reduction services. Compared to prior studies demonstrating a relationship between an individual's network size and their HCV status and risk factors [50, 51], we did not identify a relationship between HCV positivity and number of first‐degree network connections. This may be due to a number of factors. First, referrers (e.g., those with an out‐degree of 1 or more) were more likely to have received harm reduction services, including personal drug use equipment, which may have offset their HCV risk from equipment sharing. Further, we only assessed social networks through referral chains, and it may be possible that other forms of social ties, including sexual and drug use partnerships, were not necessarily represented in referral chains. This limits the ability to draw decisive conclusions about an individual's social network size, as it exists in multiple other contexts. In addition, participants who referred others may have greater social capital and ability to protect themselves from HCV infection by mechanisms including decreased use of shared supplies [52, 53]. Finally, the mean degree of our participants was one, with few higher‐degree individuals, which may have limited the ability to detect a true correlation.

This study offers unique insights into the network characteristics of rural PWUD and the relationship—or lack thereof—between these characteristics and individual HCV positivity. It highlights the heterogeneity, both with respect to HCV positivity and other characteristics, among network members. Since most participants were connected to at least one other person, interventions led by peer champions such as peer‐led HCV testing, secondary distribution of test kits and other harm reduction supplies, and involvement of peer navigators in directing people to treatment for substance use and HCV may facilitate improved access to critical services for HCV risk mitigation. This strategy is supported by our findings of peers with more referrals being more likely to engage in harm reduction services, as well as prior research identifying peer support and peer‐led testing as a means of increasing uptake and accessibility of both HCV and HIV treatment among PWUD [54, 55, 56, 57].

Given the association between HCV positivity and treatment for substance use, HCV treatment and testing should be incorporated into substance use treatment programs and harm reduction organisations to maximise outreach to PWUD who may be at higher risk of HCV positivity. Though there may be logistical and financial barriers to upscale embedded wraparound HCV services within substance use and treatment spaces [58], integrated HCV testing and treatment programs have demonstrated success in engaging PWUD in testing and treatment [59, 60]. Limited funding and coverage for non‐insured individuals remain obstacles to implementing these services, and expanded funding and coverage should be prioritised if we are to meet the goal of HCV elimination by 2030 [61].

Recognizing that HCV positivity in our sample was reported in similar proportions among isolated and non‐isolated individuals, prevention and treatment strategies should also account for those who are less socially connected and those who are not engaged in harm reduction or substance use services. To this point, it is worth noting that many of the isolated individuals who were recruited for this study were initially contacted through settings such as community outreach events and other venue‐based recruitment strategies, as opposed to being referred through the harm reduction organisation itself. Additional strategies that engage the general population, such as public health education around universal HCV testing and increasing HCV treatment among primary care providers, will be critical in addressing the epidemic in rural areas. Further, offering low‐barrier testing and treatment in a variety of sites, including mobile units and community health fairs, may be helpful in reaching those without a direct connection to a harm reduction organisation. It may also be that the isolated individuals, while not referred in by other participants in our sample, have their own peer networks that are not well‐connected with the harm reduction provider or PWUD accessing harm reduction services. Ongoing discussion with sample isolates has the potential to highlight new avenues for outreach and offers another opportunity to engage people with lived and living experience in distributing supplies and information to their community.

This study is not without its limitations. Our sample, while largely reflective of the demographic composition of the region, is predominantly white. Our method of defining network connectivity was through referral chains, and we did not capture which participants may be connected in other ways (e.g., using drugs together or sexual partnerships). Additional data on social relationships outside of the study pool was not obtained, limiting our ability to make broader inferences. While this provided valuable information around the profiles of PWUD who access harm reduction services and some of their network members, the findings should not be viewed as exhaustive. Finally, individuals who consent to participate in research studies may not be representative of the broader community of PWUD with respect to their social network structures or other characteristics.

Further study is needed to address the above limitations and explore possible avenues from implementing harm reduction strategies that leverage these findings. Researchers should investigate individual and network HCV positivity correlates among a more diverse sample and collect additional network data to better understand how different groups and sub‐groups of community members may relate to an individual's HCV status. This data should include an individual's connections to peers in a given study sample as well as social relationships and dynamics in their broader community. In addition, the data suggest that promotion of HCV prevention, screening, and treatment may be delivered by harm reduction organisations to participants with the correlated characteristics described above. Efficacy of these interventions, as well as peer‐to‐peer interventions to promote safer network drug use behaviours, should be examined in future investigations. A diverse set of approaches will be required to achieve HCV elimination in this most vulnerable population.

## Funding

This work was supported by the National Institute on Drug Abuse (UG3 DA044829, UH3 DA044829).

## Conflicts of Interest

The authors declare no conflicts of interest.

## Data Availability

The data that support the findings of this study are available from the corresponding author upon reasonable request.
